# Novel Arsenic Nanoparticles Are More Effective and Less Toxic than As (III) to Inhibit Extracellular and Intracellular Proliferation of *Leishmania donovani*

**DOI:** 10.1155/2014/187640

**Published:** 2014-12-31

**Authors:** Sudipta Chakraborty, Kaushik Bhar, Sandip Saha, Rajarshi Chakrabarti, Anjali Pal, Anirban Siddhanta

**Affiliations:** ^1^Department of Biochemistry, University of Calcutta, Kolkata 700019, India; ^2^Department of Civil Engineering, Indian Institute of Technology, Kharagpur 721302, India

## Abstract

Visceral leishmaniasis, a vector-borne tropical disease that is threatening about 350 million people worldwide, is caused by the protozoan parasite *Leishmania donovani*. Metalloids like arsenic and antimony have been used to treat diseases like leishmaniasis caused by the kinetoplastid parasites. Arsenic (III) at a relatively higher concentration (30 *μ*g/mL) has been shown to have antileishmanial activity, but this concentration is reported to be toxic in several experimental mammalian systems. Nanosized metal (0) particles have been shown to be more effective than their higher oxidation state forms. There is no information so far regarding arsenic nanoparticles (As-NPs) as an antileishmanial agent. We have tested the antileishmanial properties of the As-NPs, developed for the first time in our laboratory. As-NPs inhibited the *in vitro* growth, oxygen consumption, infectivity, and intramacrophage proliferation of *L. donovani* parasites at a concentration which is about several fold lower than that of As (III). Moreover, this antileishmanial activity has comparatively less cytotoxic effect on the mouse macrophage cell line. It is evident from our findings that As-NPs have more potential than As (III) to be used as an antileishmanial agent.

## 1. Introduction

Visceral leishmaniasis or Kala-azar, an infective disease encountered in tropical and subtropical regions of the world including Indian subcontinent, is caused by protozoan parasite,* Leishmania donovani*.* L. donovani* has two morphological forms in its life cycle: the motile flagellated slender promastigotes that stay inside the midgut of Sandfly vector and the immotile nonflagellated oval amastigote present in the phagolysosomal complex of mammalian host macrophages. The survival and proliferation of the amastigotes followed by the reinfection of new macrophages by evading the host immune machinery ensure the progression of the disease in the mammalian host. Overall annual prevalence of the disease is approximately 12 million people and the size of the population at risk is approximately 350 million. It is estimated that about 500,000 cases of visceral leishmaniasis occur worldwide each year [1–4].

Two closely related metalloids like arsenic and antimony are the drugs of choice to treat diseases caused by the kinetoplastid parasites. Antimony and arsenic are members of the same group, XV, of the periodic table and are transported into cells by the same channels, carriers [5], and pumps [6]. Interestingly, arsenic-resistant* Leishmania* species show cross resistance to antimony [7, 8]. Previously, it has been shown that 30 *μ*g/mL of sodium arsenite has antileishmanial activity [9]. However, this concentration of arsenic is reported to be toxic in several systems [10, 11]. It was found that aromatic arsenic compounds were most active against trypanosomes. The selectivity of these arsenical drugs towards trypanosomes rather than the host is apparently conferred by the aromatic ring structure [12, 13]. Promising* in vitro* trypanocidal activity was obtained with arsonoliposomes [14].

Nanomedicines have been proved to be much more effective than the conventional medicines. Nowadays, nanomedicine is rapidly evolving as an extensively studied potent therapeutic tool [15, 16]. Various exciting new works were done throughout the world in the interdisciplinary field of therapy for leishmaniasis using nanomedicine [17–20]. So far, only a few reports are known describing the usage of metal nanoparticles as antileishmanial agents [21–23]. Despite usages of Arsenic (III) form as antileishmanial agent, use of Arsenic nanoparticle (As-NPs) is yet to be reported.

In our laboratory, for the first time, stable yellowish-brown colored spherical As-NPs have been synthesized [24]. The aim of this study is to investigate whether the antileishmanial efficacy of As is enhanced in its nanoparticulate form. Here, we have tested the effect of As-NPs on the growth, oxygen consumption, infectivity, and intracellular proliferation of this parasite at a concentration which is manyfold less than that of As (III).

## 2. Materials and Methods

### 2.1. Reagents and Plasmids

Unless otherwise mentioned, all biochemicals such as FCS, Antibiotics, and G418 were of highest quality and were purchased from Sigma Chemical Co. (USA). The pTEX-EGFP plasmid was a generous gift from Dr. Martin C. Taylor, London School of Hygiene and Tropical Medicine, UK.

### 2.2. Parasite Culture

Promastigotes of* L. donovani* (MHOM/IN/83/AG83) were cultured at 23°C in RPMI 1640 media containing 10% heat inactivated FCS (fetal calf serum), 25 mM HEPES, 2 mM L-glutamine, 50 U/mL penicillin, and 50 *μ*g/mL streptomycin. Parasites were inoculated at a density of 1 × 10^6^ cells/mL and grown for 3 days to obtain exponentially growing log phase cells, which were used for subculture of the parasites.

### 2.3. Preparation and Electron Microscopy of As-NPs

Elemental grey arsenic (0), in the bulk stage (As_4_), is solid and insoluble in aqueous solution. Under ordinary condition, an oxide layer covers the core of elemental arsenic. Thus, the effect of elemental arsenic (0) is difficult to evaluate in an aqueous media; that is, why arsenic (0) cannot be applied in the cell culture medium. Arsenic (0) is made water soluble only by making it a nanoparticle. As-NPs used in this work were prepared using a simple wet-chemical procedure and characterized by SEM and TEM as published earlier [24]. Briefly, in a typical procedure, 1.0 mL of NaAsO_2_ (LOBA Chemicals) having concentration 1.0 × 10^−2 ^M was mixed with 12 mL of distilled water, and, to this solution, 0.6 mL of ice-cold NaBH_4_ solution was added having concentration 1.0 × 10^−1 ^M. The mixture was allowed to stand at room temperature for 2 hours when yellowish-brown colored As (0) solution appeared. The solution was further heated to ~60°C for 15 minutes and cooled to room temperature. The final concentration of As (0) was 735 *μ*M.

SEM, TEM, and DLS (Malvern) measurements of the As (0) solution indicated that the particles were of spherical shape, and the calculated average particle size was 76 nm (see Supplementary Figure 1 in Supplementary Material available online at http://dx.doi.org/10.1155/2014/187640). The DLS measurement was carried out in a Malvern Nano ZS instrument equipped with a 4 mW He-Ne LASER (*λ* = 632.8 nm) according to manufacturer's protocol. The zeta potential of the solution has been measured and found to be −18.1 mV, indicating that the particles are negatively charged. The detailed characterization is published earlier [24].

### 2.4. Growth Kinetics of* L. donovani* Using Different Concentration of As-NPs

Parasites were inoculated at a density of 0.5 × 10^6^ cells/mL and incubated at 23°C in 5 mL RPMI-1640 media for 24 hours. Following incubation, 0.6 × 10^6^ cells/mL were incubated further without and with different concentrations of As-NPs. Promastigote numbers were counted at an interval of 24 hours for 2 consecutive days in a Neubauer improved counting chamber (Haemocytometer) using a phase contrast microscope. The average cell numbers for each set from three independent experiments were plotted graphically. IC_50_ value was determined by plotting log of cell numbers after 24 hours of incubation with As-NPs versus concentrations of As-NPs used.

### 2.5. Cytotoxicity Assay of As-NPs and NaAsO_2_ [As (III)] on* Leishmania* Promastigotes

This assay was done according to the procedure published earlier [25]. Briefly, log phase* Leishmania* cells (1 × 10^6^ cells per each well) in 96-well plate were incubated without or with indicated concentrations of As-NPs or As (III) solution in growth media at 23°C for defined time intervals. Each concentration for every time point was done in triplicate. After the treatment, 10 *μ*L MTT solution (CCK-8, Sigma) was added to each well, following which, the readings were collected at an interval of one hour up to three hours with a microplate reader (BioRad; Model no. iMark) according to the manufacturer's protocol. The average reading of one through three hours for each set was calculated. The percentage of viable promastigotes in control and treated sets (O.D.^treated  cells^/O.D.^control^ × 100) was obtained from these average values and presented graphically. Standard errors and standard deviation were determined and plotted accordingly.

### 2.6. Cytotoxicity Assay of As-NPs on Macrophage

5000 RAW 264.7 cells were seeded in each well of a 96-well plate and incubated for 24 hours at 37°C. In the following day, these cells were treated for 24 and 48 hours with indicated concentrations of As-NPs in fresh media at 37°C. Four wells were used for each concentration for every time point. One set of untreated cells was kept as control. After the treatment, 100 *μ*L fresh media was added to each well followed by 10 *μ*L of MTT reagent (CCK-8, Sigma), following which, the readings were collected at an interval of one hour up to four hours with a microplate reader (BioRad; Model no. iMark) according to the manufacturer's protocol. The average readings of one through four hours for the sets were calculated. The quantitation of the viable cells in control and sets treated with different concentrations of As-NPs was obtained from these average values (O.D.^treated  cells^/O.D.^control^ × 100) and plotted as histogram. In a separate experiment, a standard curve was generated to determine the equivalence of the cell numbers and O.D. of MTT. *P* values were determined using paired *t*-test between untreated sets and sets treated with As-NPs.

### 2.7. EGFP Plasmid Transformation into* L. donovani* Promastigote

Transformations of empty EGFP (Enhanced Green Fluorescence Protein) plasmid into* L. donovani* promastigotes were performed by electroporation [26] with a Bio-Rad Gene Pulsar apparatus using 450 V and 550 *μ*F capacitance. Briefly, late log-phase promastigotes (0.5–1.0 × 10^7^) were harvested at 1200 g (4°C) for 10 min and washed twice in electroporation buffer (21 mM HEPES, 137 mM NaCl, 0.7 mM NaH_2_PO_4_, and 6 mM glucose, pH 7.4). Cells were finally suspended at a density of 1 × 10^8^/mL and 0.40 mL was taken into a 0.2 mm ice-chilled electroporation cuvette. Thirty micrograms of plasmid DNA dissolved in 40 *μ*L of electroporation buffer was then added to the cuvette and incubated on ice for 10 min. The cells were incubated for another 10 min on ice and added to 10 mL of drug-free growth medium (RPMI-1640). After 24 hours of revival, 20 *μ*g/mL G418 was added and the cells were kept at 26°C for another 10 days with mild shaking. The presence of transfected cells was monitored visually by microscope (Supplementary Figure 2) and the drug concentration was increased gradually with each passage. Finally, all the transfected cells were maintained in 300 *μ*g/mL of G418.

### 2.8. Measurement of Oxygen Consumption by* L. donovani* Using Gilson Oxygraph

Consumption of oxygen by parasites was measured by Gilson Oxygraph (Model: Gilson Oxygraph 5∖6) according to the procedure published earlier [27]. Parasites inoculated at a density of 1 × 10^6^ cells/mL were incubated at 37°C for 24 hours. From there, 1 × 10^6^ cells were further incubated without or with indicated concentrations of As-NPs or As (III) for defined time periods. Following incubation, 1 × 10^6^ parasite cells either treated or untreated were taken in PBS and transferred in the cell of the Oxygraph to record the oxygen consumption. The oxygen consumption was measured for 25 seconds for each set. Total oxygen dissolved in PBS inside the Oxygraph cell was measured by the addition of sodium metabisulphite and is expressed as 100%. Averages of three independent measurements of percent of oxygen consumed by the parasite cells were presented graphically. The *P* values were calculated using unpaired *t*-test.

### 2.9. Infection and Attachment of Macrophage Cells with* Leishmania* Promastigotes

RAW 264.7 cell line (ATCC TIB-71) that was used for the purpose of infection studies with* Leishmania donovani* promastigotes is a transformed murine macrophage cell line routinely used by several researchers in this field [28]. RAW 264.7 cells were seeded on to the coverslips and infected with* L.d.* parasites stably expressing EGFP (parasite/macrophage = 20 : 1). After 4 hours of incubation for internalization at 37°C, the coverslips were washed twice with media to remove nonphagocytosed promastigotes. The stock aqueous solution was diluted to the desired concentrations in respective culture media. The plate containing coverslips were then incubated without or with indicated concentrations of As-NPs or As (III) at 37°C for indicated period of time. After that, cells were fixed and stained with DAPI. Infected cells and the parasite positive dots within the infected cells were visualized in GFP and UV filters (for DAPI) in the Nikon inverted fluorescence microscope (Nikon Eclipse Ti-U). Pictures were taken in TIFF image format and were processed and merged in Adobe Photoshop CS5.

From the images, total and infected macrophage cell numbers were counted. The numbers of GFP positive punctate dots representing internalized parasites (amastigotes) were also determined. The numbers of dots per 100 infected cells thus obtained were plotted against corresponding concentrations of As-NPs and the IC_50_ value was determined accordingly from the graph.

To investigate the attachment after addition of the parasites to the macrophages, incubation was done for 4 hours at 4°C. Subsequent processing was similar to that of the infected sets.

To see the curing effect of these NPs, As-NPs (2 *μ*M) were added to the 24-hour postinfected macrophages and were further incubated for 24 and 48 hours. Numbers of total cells and average numbers of parasite positive (+) dots (from three independent experiments) within each infected cell were computed and represented graphically.

### 2.10. Statistical Analyses

Statistical significance of the results obtained was determined using the following analysis: standard deviation, paired *t*-test, unpaired *t*-test, and Fisher exact test.

## 3. Results

### 3.1. As-NPs Are More Potent in Inhibiting the Growth and Oxygen Consumption of* Leishmania* Promastigotes than As (III)

**Figure 1:**
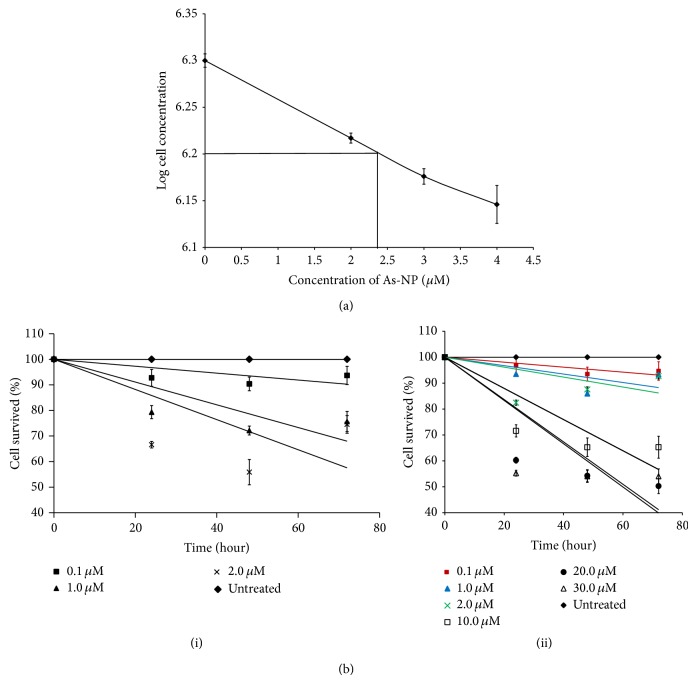
Cytotoxicity assay of* Leishmania* promastigotes by As-NPs and As (III). (a) Determination of IC_50_ value from the plot of log of promastigote cell numbers obtained after 24 hours of incubation with indicated concentrations of As-NPs. The error bars indicate SEM. (b) (i) The percentage of cell survival of* Leishmania* promastigotes treated with 0.1 μM (**■**), 1.0 μM (**▲**), and 2.0 μM (×) As-NPs for 24, 48, and 72 hours and those left untreated (**◆**) for the same time period was measured by MTT assay as mentioned in Section 2. The error bars indicate SEM. (b) (ii) The percentage of cell survival of* Leishmania* promastigotes treated with 0.1 μM (red line), 1.0 μM (blue line), 2.0 μM (green line), 10.0 μM (□), 20.0 μM (●), and 30.0 μM (△) As (III) for 24, 48, and 72 hours and those left untreated (**◆**) for the same time period was measured by MTT assay as mentioned in Section 2. The error bars indicate SEM.

The effect of different concentrations of As-NPs and As (III) on growth of* L. donovani* promastigotes was quantitatively determined by Haemocytometer under a phase contrast microscope. The IC_50_ value of the As-NP that was found to be 2.37 *μ*M was determined using the parasite cell numbers obtained from the 24-hour sample (Methods and Figure 1(a)). To further confirm our result, the cytotoxicity of As-NPs and As (III) on the promastigotes was assayed by MTT. Our results clearly demonstrated that treatment with 2 *μ*M As-NPs for 72 hours has affected the growth of promastigotes most prominently in comparison with other lower concentrations of As-NPs (0.1 and 1.0 *μ*M) (Figure 1(b)(i)). Whereas As (III) at the same range of concentration imparted minor effect on the survival of the parasites (red, blue, and green lines in Figure 1(b)(ii)), similar killing effect was only observed by treatment with 10–20 *μ*M of As (III) which is much higher than that of As-NPs (Figure 1(b)(ii)). The results of the MTT assay correlated with that of the cell counting by Haemocytometer.

**Figure 2:**
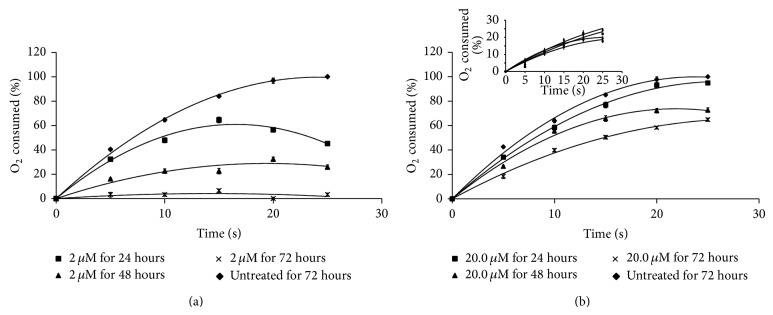
Impairment of oxygen consumption by As-NPs and As (III) treated* Leishmania* promastigotes. (a) The oxygen consumption by* Leishmania* promastigotes treated with 2 μM As-NPs for 24 (**■**), 48 (**▲**), and 72 (×) hours and those left untreated for 72 hours (**◆**) was measured by Gilson oxygraph as mentioned in Section 2. 1 × 10^6^ promastigotes were taken into the oxygraph cell and oxygen consumption was measured for 25 seconds. The average values for each set from three independent experiments were expressed as percent of the total oxygen dissolved. The data for the samples that were left untreated for 24 and 48 hours were the same as those of sample of 72 hours (not shown). (b) The oxygen consumption by* Leishmania* promastigotes treated with 20.0 μM As (III) for 24 (**■**), 48 (**▲**), and 72 (×) hours and those left untreated for 72 hours (**◆**) was measured by Gilson oxygraph as mentioned above. The percentage of oxygen consumed by these cells was graphically presented as done in panel (a) above. The “inset” shows the oxygen consumption by the parasites treated with 2.0 μM As (III) for the same time periods. The error bars indicate standard deviations in each data point in triplicate sets (*n* = 3) of experiments. The *P* values for each data point were calculated and found to be statistically significant (<0.05) (Table 1).

Having shown growth inhibitory activity on the promastigotes, both As-NPs and As (III) were tested for their effect on the parasite respiration. The respiratory activity of* L. donovani* parasites was directly assessed by measuring the percent of oxygen consumed using an oxygraph as described in Materials and Methods. Oxygen consumption by the parasites decreased gradually upon treatment with 2 *μ*M As-NPs for 24, 48, and 72 hours as compared to that by the cells left untreated (Figure 2(a)). However, as expected, the parasites treated with 2 *μ*M As (III) showed almost no effect in their oxygen consumption (Figure 2(b) inset). Even 10-fold higher concentration of As (III) was shown to have much less effect on the respiration of promastigotes (Figure 2(b)). The effects of As-NPs and As (III) on the oxygen consumption by the parasites were found to be statistically significant (Tables 1(a) and 1(b)).

### 3.2. As-NP Is More Effective than As (III) in Reducing Infectivity of* Leishmania* Promastigote* In Vitro*

**Figure 3:**
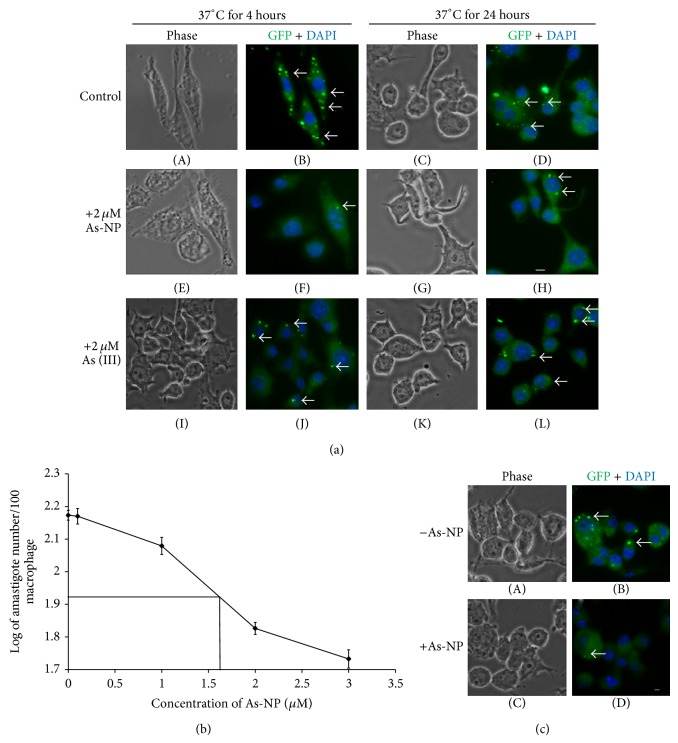
As-NPs treated parasites show reduced infectivity* in vitro*. (a) Infection of RAW 264.7 macrophages with* Leishmania* promastigote was carried out as described in Section 2.* Leishmania* infected macrophages were incubated at 37°C for 4 hours (panels (A), (B), (E), (F), (I), and (J)) and 24 hours (panels (C), (D), (G), (H), (K), and (L)) in absence (panels (A), (B), (C), and (D)) and in presence (panels (E), (F), (G), and (H)) of As-NPs (2 μM) and in presence of 2.0 μM As (III) (panels (I), (J), (K), and (L)) as described earlier, after which cells were prepared for fluorescence microscopy. Pictures were taken in phase (panels (A), (C), (E), (G), (I), and (K)), GFP filter (green), and DAPI filter (blue). The GFP and DAPI images were merged (panels (B), (D), (F), (H), (J), and (L)). Arrows indicate internalized parasites. Scale bar indicates 2 μm. These images only represent one of multiple independent sets. (b) Determination of IC_50_ value from the plot of log (internalized cells or amastigotes per 100 infected macrophages) was obtained after 24 hours of incubation with indicated concentrations of As-NPs. The error bars indicate SEM. (c) Treatment with As-NPs reduced macrophage attached* Leishmania* parasites. Attachment of* Leishmania* parasite on macrophages was determined by incubating the* Leishmania* infected macrophages at 4°C for 4 hours in absence (panels (A) and (B)) and presence (panels (C) and (D)) of As-NPs. Fluorescent pictures were captured in phase (panels (A) and (C)), GFP filter (green), and DAPI filter (blue). Panels (C) and (D) show merged images captured by GFP and DAPI filters (panels (B) and (D)). Arrows indicate macrophage attached parasites. These images only represent one of multiple independent sets. Scale bar indicates 2 μm.

The inhibitory effects of As-NPs on the growth and metabolic activity of the parasites led us to investigate their effect on the infectivity of* Leishmania* parasites* in vitro*. We have also compared the effect of As-NPs and As (III) on the infectivity of promastigote. The macrophages that were infected with promastigotes in presence of As-NPs had modest number of GFP positive puncta (Figure 3(a); panels (F) and (H)) as compared to the set without As-NPs indicating reduction of parasite burden in the macrophages (Figure 3(a); panels (B) and (D) and Table 2(a)). However, in presence of As (III), the numbers of internalized parasites (amastigotes) were very similar to those found in control sets (Figure 3(a); compare panels (J), (L) and (B), (D) with (F), (H)). IC_50_ values that were determined using range of concentrations of As-NPs in the infection studies (37°C for 24 hours of incubation) were found to be 1.5 *μ*M (Figure 3(b)). Moreover, the number of fluorescent parasites attached on macrophage surface was also reduced by the treatment with the nanoparticles indicating an inhibition in the attachment of the parasites to the macrophages prior to internalization (Figure 3(c); compare panels (B) and (D) and Table 2(b)). These experiments were repeated several times and resulted in very similar observation.

### 3.3. As-NPs Blocked Intramacrophage Proliferation of* Leishmania* Parasites

**Figure 4:**
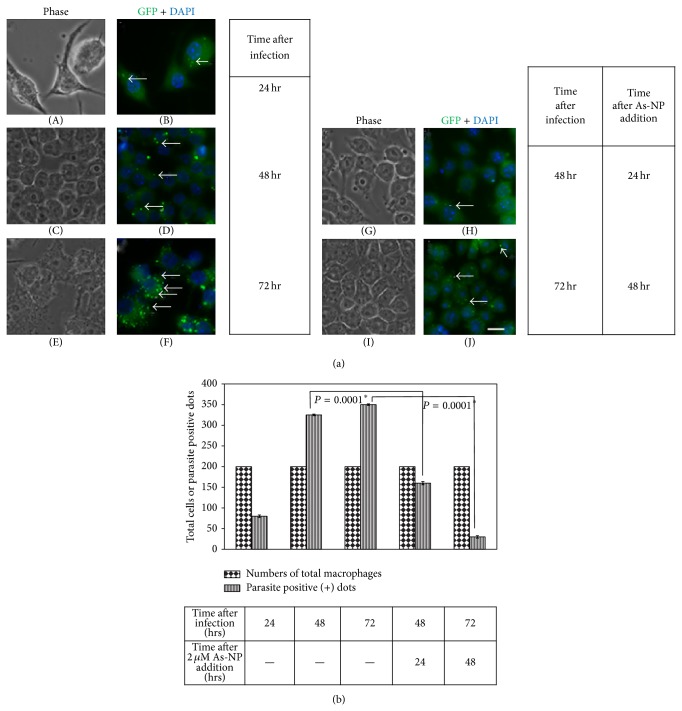
As-NPs blocked intramacrophage proliferation of* Leishmania* parasites. (a) As-NPs reduced the postinfection proliferation of the parasites inside the macrophages. RAW macrophages were infected with* Leishmania* promastigotes and incubated for 24 hours at 37°C. 24-hour postinfected macrophages (panels (A) and (B)) were further incubated without ((C)–(F)) and with ((G)–(J)) As-NPs for another 24 ((C), (D), (G), and (H)) and 48 ((E), (F), (I), and (J)) hours. Phase ((A), (C), (E), (G), and (I)), GFP (green), and DAPI (blue) pictures were taken in fluorescence microscope and the GFP and DAPI images were merged ((B), (D), (F), (H), and (J)). These images only represent one of multiple independent sets. Scale bar indicates 2 μm. (b) Quantitation of the intracellular proliferation of* L. donovani* promastigotes in 24-hour postinfected macrophages incubated in presence of 2 μM As-NPs for another 24 and 48 hours. Numbers of total macrophage and parasite positive (+) dots within each infected cell were separately determined by manual counting of representative images and presented graphically. The error bars indicate standard deviations for the number of parasite positive dots obtained from three independent experiments. ∗ denotes statistically significant *P* values (<0.05).

In the preceding section, we have demonstrated the suppressive effect of As-NPs on the attachment and infectivity of the parasite. Furthermore, we asked whether the nanoparticles can exert effect on the postinfection proliferation of the parasites inside the macrophages. To address that, 24-hour postinfected macrophages were treated with As-NPs for another 24 and 48 hours (Figure 4(a); panels (G), (H) and (I), (J), resp.). Our results clearly showed that As-NPs can also block parasite proliferation inside the macrophage significantly (Figure 4(a): compare panels (D), (F) with (H), (J) and Figure 4(b)). While the parasite positive fluorescent puncta in As-NP untreated cells increased significantly over time (Figure 4(a); panels (B), (D), and (F)), the parasite positive dots in the infected cells that were treated remained static (Figure 4(a): compare panels (B), (D), and (F) with (H) and (J) and Figure 4(b): in treated samples: parasite positive dots). Repeated addition of fresh As-NPs after 24 hours of the first addition did not yield much effect (data not shown). The result of this experiment is satisfactorily reproducible and the *P* values for this experiment that were determined by Fisher exact test are statistically significant (Figure 4(b)).

**Figure 5:**
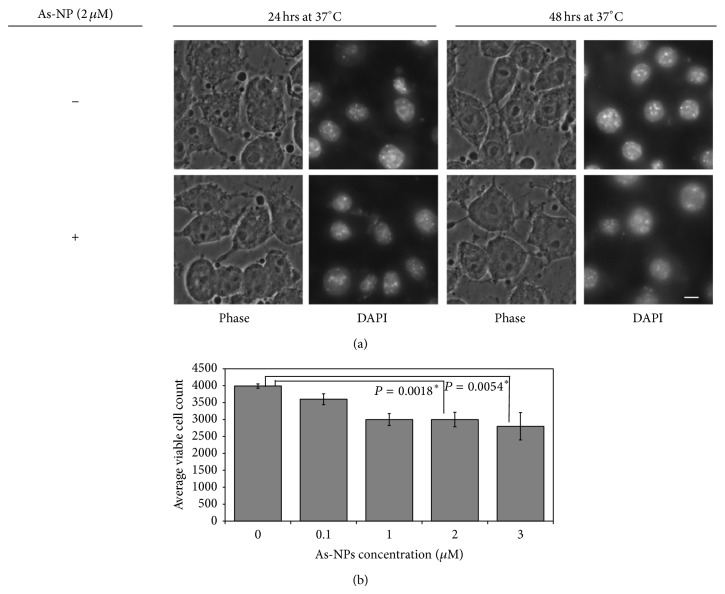
Effect of As-NPs on macrophages. (a) RAW cells were incubated without and with As-NPs (2 μM) for 24 and 48 hours at 37°C as indicated, following which, cells were stained with DAPI. Phase and DAPI images for each set are shown. These images are representative of several images obtained from multiple independent experiments. (b) The number of the viable cells in control (0) and sets treated with indicated concentrations of As-NPs was plotted. The number of the viable cells was calculated as mentioned in Section 2. In a separate experiment, a standard curve was generated to determine the equivalence of the cell numbers and the O.D. values. The number of viable cells in the untreated control set (~4000) is less than that which was seeded initially (5000). Each column with error bar represents average ± SD, *n* = 3. ∗ denotes *P* values (<0.05) of statistical significance.

Here, to explore the possibility whether the As-NPs treatment had any effect on the macrophage itself, several microscopic images of nanoparticle treated macrophages were captured.  Figure 5(a) shows representative of such images which clearly indicate that there is no change in the morphology (phase and DAPI) of macrophages treated with As-NPs. Additionally, we have checked the viability of the macrophages treated with the nanoparticles by MTT assay.  Figure 5(b) shows that As-NPs treatment has resulted in 25% loss in viable cells as compared to the untreated control. It is to be noted that the viable cell number in the control set (~4000) showed a reduction from the initially seeded cell number (5000) due to loss during the processing required for this experiment.

## 4. Discussion

Elemental Arsenic (0) is insoluble in aqueous media. It exists in nature mostly as two biologically relevant oxidation states: arsenate [As (V)] and arsenite [As (III)]. In proteins, As (III) binds to both cysteine and histidine residues and causes depletion of intracellular glutathione. In the pentavalent form, arsenic is known to compete with phosphate, particularly in the citric acid cycle to alter the production of ATP [29]. Several studies on the antibacterial, antiviral, and antifungal activities of arsenic have been carried out [30]. According to a previous report, 30 *μ*g/mL of As (III) (equivalent to about 230 *μ*M) is shown to be antileishmanial [9]. This concentration of As is very toxic in mammalian system [10, 11]. The present study is the first to demonstrate that the novel As-NPs possess better antileishmanial efficacy than that of As (III). It is noteworthy that the concentration at which the As-NPs imparted significant inhibitory effect on the viability, oxygen consumption, and infectivity of the parasite is too low for As (III) to show any significant inhibitory effect on the parasites (Figures 1–4). Moreover, the antileishmanial effect comparable to that of As-NPs is only achieved by at least 10-fold concentrated As (III). IC_50_ value determination shows that the amastigotes are more sensitive to the As-NPs than the promastigotes. This data indicates that the As-NPs will be equally effective as an antileishmanial agent* in vivo*. Furthermore, the negatively charged nanoparticles as indicated by the zeta potential value would possibly face no problem to reach the phagolysosomes of macrophages in the infected internal organs, namely, liver and spleen. While the cellular and nuclear morphologies of the host macrophage cells treated with As-NPs remain unchanged (Figure 5(a)), the viability of the treated macrophages was reduced only by 25% (Figure 5(b)). It is noteworthy that 1 *μ*M As (III) was shown to have severe toxicity* in vitro* and* in vivo* [10, 11]. Moreover, it is not intuitive to predict the extent of its toxicity* in vivo* from the* in vitro* observation. There are reports where it was shown that the levels of toxicity between these two systems vary significantly [10, 11]. Taken together, our findings suggest that the As-NPs have very good potential as compared to the As (III) to be developed as an effective antileishmanial agent with much less effect on the host macrophages.

## 5. Conclusion

Visceral leishmaniasis is a fatal disease affecting about 500,000 cases each year worldwide. It is evident that the pathogenesis depends on the proliferative capacity of the parasite inside the macrophages. If, somehow, this proliferation can be controlled, the pathogen can be rendered ineffective. The main problem of the conventional antileishmanial drugs for leishmaniasis is their toxicity and high prices. So, the development of new drug(s) which would be cheaper with lesser side effects is a long lasting requirement. Arsenic (III) at a relatively higher concentration (30 *μ*g/mL) has been shown to have antileishmanial activity; but this concentration is reported to be toxic in several experimental mammalian systems. We have shown that the As-NPs, developed for the first time in our laboratory, significantly inhibited the oxygen consumption and intracellular proliferation of the parasitic pathogen. The concentration of As-NPs is about several fold lower than that of As (III). Moreover, this antileishmanial activity has comparatively less cytotoxic effect on the mouse macrophage cell line. Thus, it is evident from our findings that As-NPs have more potential than As (III) to be developed as a cheap and less toxic antileishmanial agent.

## Supplementary Material

Figure 1. The As-NPs were characterized by DLS measurement in a Malvern Nano ZS instrument equipped with a 4mW He-Ne LASER (λ= 632.8 nm). The calculated average particle size of As-NPs was found to be 76  nm
Figure 2. *Leishmania donovani* promastigotes stably expressing EGFP were generated by transfection of pTEX-EGFP plasmid into the log phase cells using an electroporator. The image of such a promastigote stably expressing EGFP was given below

## Figures and Tables

**Table:** (a) For As-NPs

Treatment duration (h)^*^	% O_2_ of consumption (mean)^a^		*P* values^b^
	Time (s)		
24	5	4.616	0.023
	10	6.916	0.023
	15	9.340	0.0394
	20	8.140	0.0035
	25	6.496	0.0015

48	5	2.316	0.023
	10	3.253	0.0183
	15	3.246	0.0003
	20	4.643	0.0004
	25	3.726	0.0004

72	5	0.462	0.0001
	10	0.456	0.0001
	15	0.953	0.0001
	20	0.004	0.0001
	25	0.425	0.0001

^a^Mean values of percent of O_2_ consumed by *L.d.* parasites at every 5 sec up to 25 sec, in presence of 2 *µ*M As-NPs for indicated time^*^ obtained from three independent experiments.

^b^It signifies *P* values <0.05.

**Table:** (b) For As (III)

Treatment duration (h)^*^	% O_2_ of consumption (mean)^a^		*P* values^b^
	Time (s)		
24	5	7.356	0.023
	10	12.625	0.028
	15	16.640	0.0294
	20	20.140	0.0135
	25	20.496	0.0215

48	5	5.756	0.023
	10	12.053	0.0203
	15	14.216	0.0013
	20	15.603	0.0104
	25	15.716	0.0124

72	5	4.062	0.0031
	10	8.606	0.0201
	15	10.053	0.0101
	20	12.614	0.0031
	25	14.022	0.0231

^a^Mean values of percent of O_2_ consumed by *L.d.* parasites at every 5 sec up to 25 sec, in presence of 20 *µ*M As (III) for indicated time^*^ obtained from three independent experiments.

^b^It signifies *P* values <0.05.

**(a):** 

Sets	Total number of macrophages	Numbers of infected macrophages	Numbers of internalized parasites inside the macrophages
Control	500	480^*^	652^*^
2 *µ*M As-NP	500	125^*^	165^*^
0.1 *µ*M As (III)	500	435^*^	643^*^
1.0 *µ*M As (III)	500	275^*^	325^*^
2 *µ*M As (III)	500	428^*^	558^*^

^*^ *P* values for number of infected macrophages and internalized parasites are 0.001 which is statistically significant.

**(b):** 

Sets	Total number of macrophages	Numbers of infected macrophages	Numbers of attached parasites in the surface of macrophages
Control	200	52^#^	68^#^
2 *µ*M As-NP	200	22^#^	23^#^

^#^ *P* values for number of infected macrophages and attached parasites are 0.0014 which is statistically significant.

## Abbreviations

As: Arsenic
As-NPs: Arsenic nanoparticle
*L.d*.: * Leishmania donovani*
SEM: Standard error mean.
