# Supplementary material for: Novel Arsenic Nanoparticles Are More Effective and Less Toxic than As (III) to Inhibit Extracellular and Intracellular Proliferation of Leishmania donovani
Source: J Parasitol Res. 2014 Dec 31;2014:187640. doi: 10.1155/2014/187640 (PMC4295593; doi:10.1155/2014/187640)
Supplement: Supplementary file 1 — Figure 1. The As-NPs were characterized by DLS measurement in a Malvern Nano ZS instrument equipped with a 4mW He-Ne LASER (λ= 632.8 nm). The calculated average particle size of As-NPs was found to be 76 nm Figure 2. Leishmania donovani promastigotes stably expressing EGFP were generated by transfection of pTEX-EGFP plasmid into the log phase cells using an electroporator. The image of such a promastigote stably expressing EGFP was given below [file 187640.f1.doc]

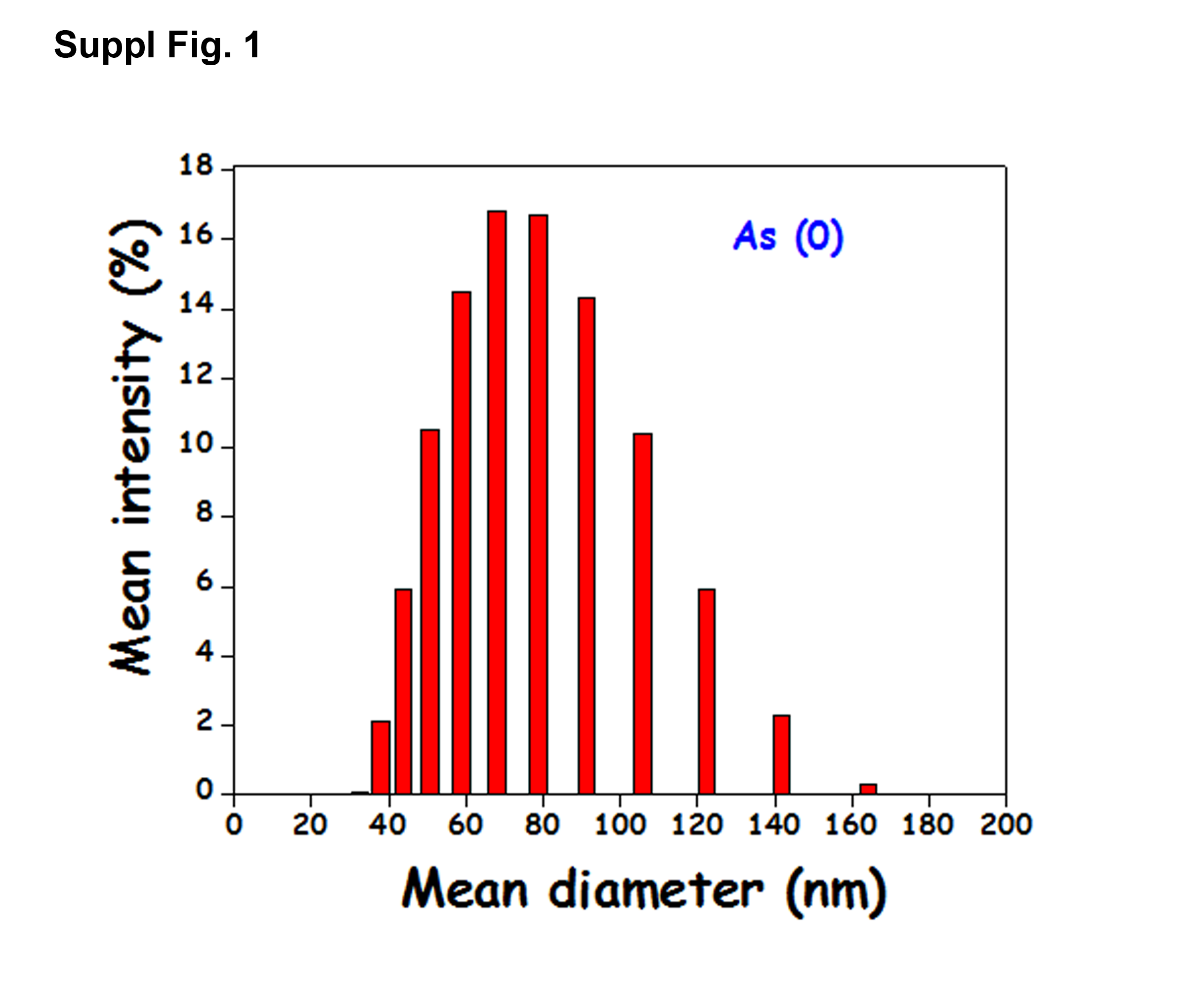


**Supplementary Fig. 1 Dynamic Light Scattering meassurement of As-NPs:** The diagram represents the statistical histogram (% of Mean intensity against mean diameter in nm)analysis of As nanoparticles**.** Histogram represents the statistics of five separate measurements ineach case.


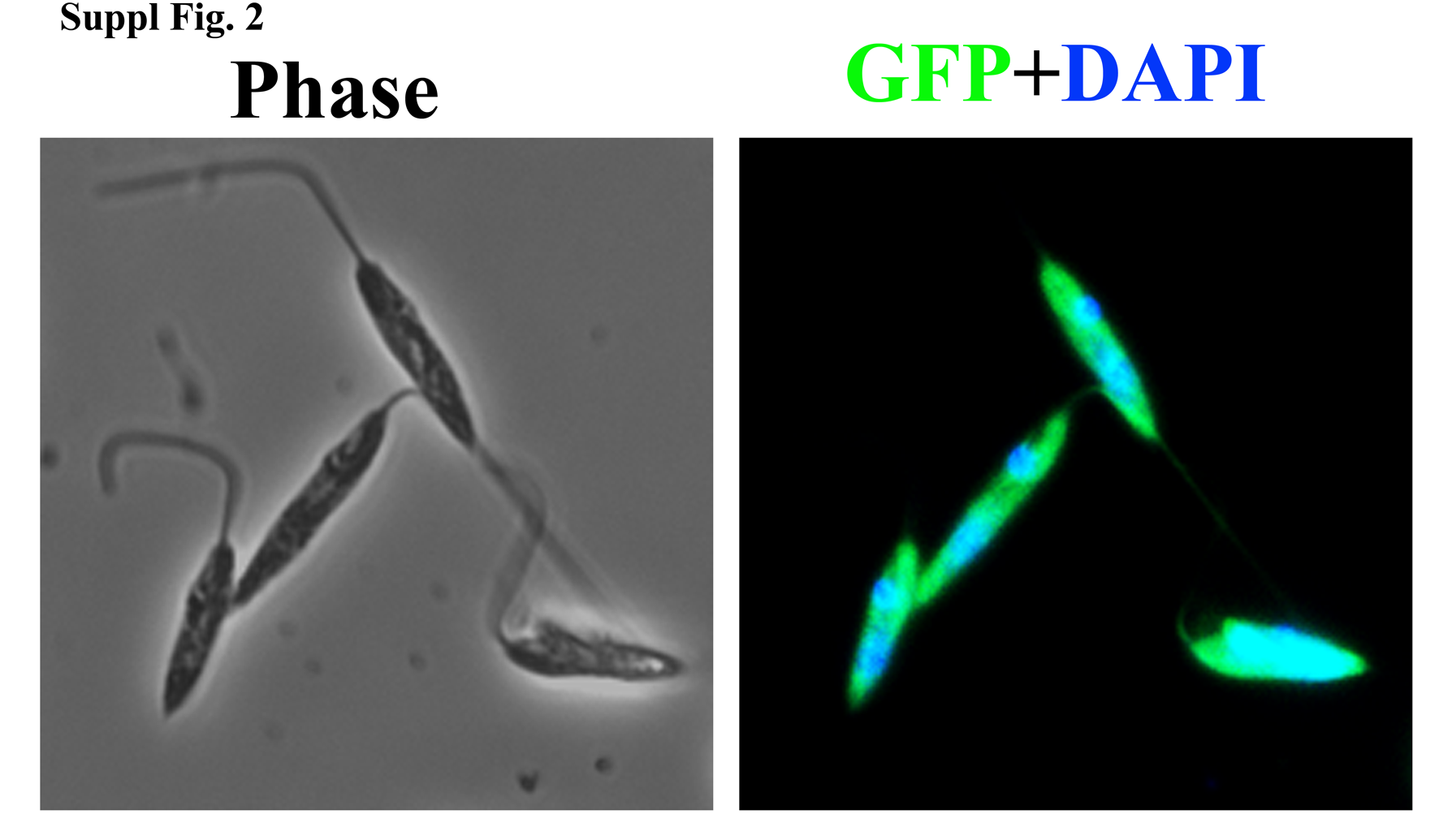


**Supplementary Fig. 2 *Leshmania* promastigotes stably expressing EGFP**

*Leishmania donovani* promastigotes were transfected with pTEX-EGFP plasmid by electroporation as described in the ‘Materials and Methods’. The promastigotes stably expressing EGFP were selected by G418 treatment. The stable cells were stained with DAPI to visualize nuclei and fluorescent microscopic pictures were captured in ‘Phase’, GFP (green) and UV (blue) filters. The green and blue images were merged (GFP+DAPI) using ADOBE PHOTOSHOP CS5.
